# Genetics of amyotrophic lateral sclerosis: an update

**DOI:** 10.1186/1750-1326-8-28

**Published:** 2013-08-13

**Authors:** Sheng Chen, Pavani Sayana, Xiaojie Zhang, Weidong Le

**Affiliations:** 1 Institute of Neurology, Jiao Tong University School of Medicine, 1201 Room, 11 Building, Ruijin Er Road, Shanghai 200025, China; 2 Department of Neurology, Baylor College of Medicine, Houston, TX 77030, USA

**Keywords:** Amyotrophic lateral sclerosis, Disease-related gene mutations, Autophagy, Apoptosis, Oxidative stress, Glutamate excitotoxicity

## Abstract

Amyotrophic lateral sclerosis (ALS) is a progressive neurodegenerative disorder involving both upper motor neurons (UMN) and lower motor neurons (LMN). Enormous research has been done in the past few decades in unveiling the genetics of ALS, successfully identifying at least fifteen candidate genes associated with familial and sporadic ALS. Numerous studies attempting to define the pathogenesis of ALS have identified several plausible determinants and molecular pathways leading to motor neuron degeneration, which include oxidative stress, glutamate excitotoxicity, apoptosis, abnormal neurofilament function, protein misfolding and subsequent aggregation, impairment of RNA processing, defects in axonal transport, changes in endosomal trafficking, increased inflammation, and mitochondrial dysfunction. This review is to update the recent discoveries in genetics of ALS, which may provide insight information to help us better understanding of the disease neuropathogenesis.

## Background

Amyotrophic lateral sclerosis (ALS) is a heterogeneous group of neurodegenerative disorders characterized by progressive loss of motor neurons of the primary motor cortex, brainstem and spinal cord, consequently resulting in muscle weakness, paralysis and ultimately the death [1]. Patients present with either limb onset (80% cases) or bulbar onset (20% cases). In limb onset cases, symptoms appear either distally or proximally in either the upper or lower limb. Bulbar onset cases usually manifest with dysarthria and dysphagia, and limb symptoms can develop along with bulbar symptoms or may occur in the due course of the disease within a year [1]. Most ALS patients come across both upper motor neuron (UMN) and low motor neuron (LMN) signs. The typical age onset is about 55 years [2]. It progresses at a fast pace with most of the patients dying within 3–5 years of the onset [2]. However there is also a small subset of ALS cases that present with a relatively slower disease course. The incidence of the disease is approximately similar worldwide ranging from 1 to 2 new cases per 100,000 individuals every year and the prevalence is around 4–6 cases per 100,000 individuals [2].

ALS has no definitive diagnostic test and it is diagnosed clinically in most cases. There is no cure for ALS, although the only FDA approved drug Riluzole may increase certain ALS patient survival by 3 months [3]. Considerable progress has been made in comprehending the genetics of ALS in the past decade. Interestingly, recent studies have demonstrated that ALS may be a multisystem neurodegenerative disease in which brain and brain stem are also affected [1-3]. For example, several cases suffered from ALS exhibit symptoms of cognitive impairment, which is pathologically characterized by cytoplasmic ubiquitin-positive inclusions in hippocampus and neocortical neurons in addition to anterior horn neurons [1-3]. In this review, we summarize the genetic breakthroughs in familial ALS (fALS) and sporadic ALS (sALS) and depict how it shapes our understanding of disease pathogenesis and our quest for rational therapies.

## Genetics of ALS

**Figure 1:**
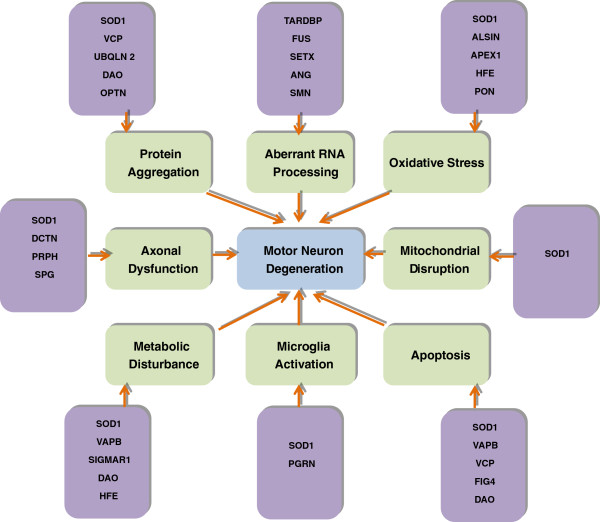
**ALS is caused by interplay of various molecular pathways in motor neurons and an interaction with neighbouring non-neuronal cells like microglia and astrocytes.** Microglial cells activate an inflammatory cascade via secretion of cytokines. Astrocytes lead to motor neuron injury through release of inflammatory mediators such as nitric oxide and prostaglandin E2. Accumulation of superoxide radicals and oxidative stress, aberrant RNA processing, protein misfolding and insoluble proteins may cause motor neuron degeneration in ALS. Protein aggregation may lead to endoplasmic reticulum stress along with defective endosomal trafficking and mitochondrial damage, which may cause organelle disruption and activates autophagy and apoptotic pathways. Axonal transport abnormalities lead to energy deficiency in the axon along with the defective axonal growth and axonal dysfunction. Axonal dysfunction, defective angiogenesis and metabolic disturbance may contribute to motor neuron degeneration in ALS.

ALS can be inherited in an autosomal dominant, autosomal recessive, or X-linked manner. 90% ALS are sporadic cases with no clear genetic linkage. However, the remaining 10% of cases show familial inheritance [4,5]. In the last few years, there has been a rapid advance in our knowledge of genetic causes for ALS. Furthermore, the relationship between the genetic subtypes and the pathological subtypes as well as clinical phenotype has become more and more clear. In addition to superoxide dismutase 1 (SOD1), mutations in the genes coding for TAR DNA-Binding Protein (TARDBP), fused in sarcoma (FUS), Ubiquilin2 (UBQLN2), C9ORF72 and several others are closely associated with typical clinical phenotype. Figure 1 provides up-to-date findings of genetic defects in ALS and the underlying mechanisms for the cause and pathogenesis of the disease.

### Genetics of fALS

The causative genes have been identified in almost 5-10% of all fALS cases to date [4,5]. Among those 20% of fALS cases are caused by the mutation in SOD1 gene, 4-5% of fALS cases are the results of mutations in TARDBP and FUS genes, more than 30% of fALS cases are associated with C9ORF72 mutations and the rest are due to the mutations in alsin, senataxin (SETX), spatacsin, vesicle associated membrane protein associated protein B (VAPB). angiogenin (ANG), factor induced gene 4 (FIG 4), optineurin (OPTN) and perhaps other unknown genes [see Table 1].

**Table 1:** The genetics Of fALS

**Genetic subtype**	**Chromosomal locus**	**Gene**	**Protein**	**Onset**	**Inheritance**	**Clinical feature**	**Other diseases caused by the gene**
ALS1	21q22.1	SOD1	Cu/Zn SOD-1	Adult	AD/AR	Typical ALS	NA
ALS2	2q33-2q35	Alsin	Alsin	Juv	AR	Slowly progressive, predominantly UMN signs like limb, & facial spasticity	PLS IAHSP
ALS3	18q21	Unknown	Unknown	Adu	AD	Typical ALS with limb onset especially lower limb	NA
ALS4	9q34	SETX	Senataxin	Juv	AD	Slowly progressive, distal hereditary motor neuropathy with pyramidal signs	SCAR 1 and AOA2
ALS5	15q15-21	SPG 11	Spatacsin	Juv	AR	Slowly progressive	HSP
ALS6	16p11.2	FUS	Fused in Sarcoma	Juv/Adu	AD/AR	Typical ALS	NA
ALS8	20q13.3	VAPB	VAPB	Adu	AD	Typical and atypical ALS	SMA
ALS9	14q11.2	ANG	Angiogenin	Adu	AD	Typical ALS, FTD and Parkinsonism	NA
ALS10	1p36.2	TARDBP	DNA-binding protein	Adu	AD	Typical ALS	NA
ALS11	6q21	FIG 4	Phosphoinositide-5phosphatease	Adu	AD	Rapid progressive with prominent corticospinal tract signs	CMT 4 J
ALS12	10p13	OPTN	Optineurin	Adu	AD/AR	Slowly progressive with limb onset and predominant UMN signs	Primary Open Angle Glaucoma
ALS14	9p13.3	VCP	VCP	Adu	AD	Adult onset, with or without FTD	IBMPFD
ALS15/ALSX	Xp11	UBQLN2	Ubiquilin 2	Adu/Juv	XD	UMN signs proceeding LMN signs	NA
ALS16	9p13.2-21.3	SIGMAR1	SIGMAR1	Juv	AR	Juvenile onset typical ALS	FTD
ALS-FTD1	9q21-22	unknown	unknown	Adu	AD	ALS with FTD	FTD
ALS-FTD2	9p21	C9ORF72	C9ORF72	Adu	AD	ALS with FTD	FTD
NA	2p13	DCTN1	Dynactin	Adu	AD	Distal hereditary motor neuropathy with vocal paresis	NA
**Other rare-occurring ALS genes**							
ALS3	18q21	Unknown	Unknown	Adu	AD	Typical ALS with limb onset especially lower limb	NA
ALS7	20ptel-p13	Unknown	Unknown	Adu	AD/AR	Typical ALS	NA
NA	12q22-23	DAO	DAO	Adu	AD	Typical ALS	NA

Up to date, more than 20-ALS genes have been identified in fALS. These genetic mutations represent different molecular pathways of motor neuron degeneration.

Abbreviations for Table 1: *PLS* Primary Lateral Sclerosis, *IAHSP* Infantile onset ascending hereditary spastic paralysis, *SCAR 1* Autosomal Recessive Spino-cerebellar ataxia, *AOA2* Ataxia Ocular Apraxia 2, *HSP* Hereditary spastic paraplegia, *VAPB* Vesicle associated membrane protein associated protein B, *SMA* Spinal Muscular Atrophy, *CMT 4 J* Charcot-Marie Tooth disease type 4 J, *VCP* Valosin Containing Protein, *IBMPFD* Inclusion body myopathy with Pagets disease and fronto temporal dementia, *SIGMAR1* Sigma Non Opiod Intracellular Receptor, *C9ORF72* Chromosome 9 open reading frame 72, *PD* Parkinson disease, *DAO* D-Amino Acid Oxidase, *FTD* Frontal-temporal dementia, *AD* Autosomal dominant, *AR* Autosomal recessive.

#### ALS1/ superoxide dismutase 1(SOD1)

SOD1 is the first gene to be identified in fALS based on linkage analysis in autosomal dominant fALS pedigrees, which maps to chromosome 21q22.1 [5]. Individuals with mutant SOD1 present mostly with limb onset, starting predominantly in lower limb rather than upper limb. A few cases also present with bulbar onset. To date more than 150 mutations have been found in all 5 exons affecting the functional domains of SOD1 predominantly in missense mutations, although a small percentage of nonsense mutations, insertions and deletions have also been reported. Mutations in SOD1 have been reported in ~20% of fALS and in ~1-4% of sALS [2]. SOD1 mutant ALS cases show ample variation in the phenotype in the age of onset, severity, rate of disease progression and duration. SOD1 genotype analysis illustrates variable clinical phenotypes, indicating that the phenotype is modified by genetic as well as environmental factors [4]. The SOD1^D90A^ mutation which is recessive in a Scandinavian population is shown to be related with autosomal dominant ALS in other genetic groups [2]. Patients carrying the SOD1^A4V^ mutations have shorter survival with death occurring in less than one and half year after the diagnosis, and the penetrance of the gene mutation is 91% [2]. The SOD1^A89V^ mutation has incomplete penetrance, variable age at onset and sensory neuropathy [6]. The SOD1^I113T^ mutation is highly diverse in the age of onset, clinical manifestations, disease progression and penetrance [7,8].

SOD1 is a cytoplasmic protein, which is a homodimer of 153 amino acids, containing one copper and one zinc atom [2,5]. Copper plays a role in SOD1 activity, whereas zinc is contributed to structural stability. SOD1 detoxifies superoxide radicals, a by-product of oxidative phosphorylation, to hydrogen peroxide and oxygen, which are converted to water and oxygen by catalase and glutathione peroxidase enzymes [2,5]. The exact mechanism by which SOD1 mutations lead to ALS pathology is unknown although numerous hypotheses have been proposed to explain SOD1-mediated toxicity such as misfolding proteins-associated aggregation, oxidative stress, mitochondrial dysfunction, endoplasmic reticulum stress, glutamate excitotoxicity, inflammation and microglial activation and axonal transport abnormalities [2,5]. Several mutant SOD1 transgenic mice have been generated and SOD1^G93A^ mice are the mostly used model for ALS studies [9]. This mouse model has been successfully used in variety of studies to define the molecular mechanism of the disease and evaluate new drug responses [9]. Interestingly, SOD1 knockout mice do not develop ALS, although they do exhibit some age-dependent distal motor neuropathy compared with SOD1^G93A^ mice, which supports the notion that toxic gain of function may be responsible for the motor neuron degeneration in SOD1^G93A^ mice since enzymatic activity is retained in SOD1^G93A^ mutation [10].

#### ALS2/ALSIN

ALS2 is a rare, autosomal recessive, juvenile onset (mean age at onset is 6.5 years) disease characterized by limb and facial spasticity, spastic dysarthria, uncontrolled laughter, subsequent lower motor neuron signs and bladder dysfunction [2,11]. The locus of ALS2 is mapped to chromosome 2q33-2q35 via linkage analysis in a large Tunisian kindred [2,11]. The deletion mutations result in frame shifts that result in a premature stop codon. Alsin is alternatively spliced to produce a long and a short transcript. Deletion mutations in the short transcript cause ALS2 and those in the long transcript lead to juvenile primary lateral sclerosis (PLS) [11]. Alsin is a Rab5 and Rac1 guanine nucleotide exchange factor (GEF) domains. It promotes neurite outgrowth in cell cultures through activation of the small GTPase Rac1 macropinocytosis-associated endolysosomal trafficking via the fusion between endosomes and autophagosomes [11]. Alsin protects cultured motor neurons from mutant SOD1 toxicity suggesting its neuroprotective role [12]. Overexpression of alsin inhibits mutant SOD1^G93A^-induced endosomal Rac1 activation and reactive oxygen species production [13,14]. The mutations in alsin may induce a loss of this neuroprotective function by disrupting the endolysomal system and causing an aggregation of immature vesicles and misfolded proteins in neurons [13,14]. Alsin knock-out mice models demonstrate that the loss of alsin can result in motor neuron damage, but no definitive features consistent with ALS or other motor neuron disease. However, not all the studies support that alsin knock-out mice might develop motor neuron degeneration. Cai et al. and Gros-Louis et al. reported that alsin knock-out mice displayed signs of corticospinal track degeneration but did not develop obvious progressive motor neuron degeneration [15,16]. These mice have increased vulnerability to oxidative stress, indicating that alsin variants might be a risk factor rather than directly cause of motor neuron degeneration [15,16].

#### ALS4/senataxin (SETX)

ALS4 is a rare, juvenile onset, autosomal dominant ALS, characterized by distal limb weakness, muscle atrophy, and pyramidal signs [1,2,17]. Bulbar and respiratory muscles are spared. Disease progression is slow and the patients usually have a normal life span [1,2,17]. ALS4 is linked to chromosome 9q34 [17] and sequencing 19 genes in this locus reveals 3 distinct missense mutations in the SETX gene in 3 families with ALS [18]. In addition, positional cloning technique reveals that the mutations in SETX gene in the locus also associated with autosomal recessive spinocerebellar ataxia-1 (SCAR1), which is also referred to ataxia-ocular apraxia-2 (AOA2) [19]. SETX gene encodes a ubiquitously expressed DNA/RNA helicase protein [19,20]. SETX and DNA/RNA helicases are involved in DNA repair, replication, recombination, transcription, RNA processing, transcript stability, and the initiation of translation [19,20]. SETX is similar to several genes involved in RNA processing such as the immunoglobulin mu binding protein 2 gene (IGHMBP2), mutations in which are known to cause spinal muscular atrophy with respiratory distress type 1 [20], implying that SETX mutations-caused motor neuron degeneration may result from the aberrant RNA processing [21].

#### ALS5/spatacsin (SPG)

ALS5 also known as juvenile ALS type 1 is the most common form of recessive fALS with juvenile onset. Most of the cases present before the age of 25 yrs [22,23]. It follows a slow progressive course that can survive more than 3 decades. A study in 22 patients with ALS from 7 families links ALS5 to chromosome 15q15-21 and mutations in SPG11 gene [22,23]. SPG11 gene mutations cause autosomal recessive hereditary spastic paraplegia (HSP) with atrophied corpus callosum [22,23], and share certain clinical, pathological and genetic features of ALS according to a study in 25 families from Italy, Brazil, Canada, Turkey, and Japan [24]. A total of 12 mutations in SPG11 gene have been identified, out of which 10 are either nonsense mutations or insertions or deletions leading to a frame shift, indicating a loss of function [24]. SPG11 is a protein with four transmembrane domains, a leucine zipper and a coil domain. This protein is identified in central nervous system, especially in the cortical and spinal motor neurons as well as in the retina, showing multiple organelles like protein-trafficking vesicles, endoplasmic reticulum and microtubules, and the accumulation of spatacsin in non-myelinated axons suggesting axonal transport disturbance [25].

#### ALS6/fused in sarcoma (FUS)

ALS patients with ALS6 mutations are characterized by a wide range of disease onset from 26–80 years with the mean duration around 33 months [2]. Most cases show LMN predominance, without bulbar region involvement and no cognitive impairment. The locus for ALS6 has been mapped to chromosome 16p11.2 encoding FUS gene [26]. Mutations in FUS gene are identified based on a linkage analysis in a large Cape Verde family with autosomal recessive ALS and in numerous British kindred with autosomal dominant ALS [27]. Up to now, more than 50 FUS mutations have been identified in ~4% cases of fALS and ~1% of sALS cases [27]. FUS mutations are also found in fALS patients with frontal-temporal dementia (FTD) and in juvenile ALS patients with basophilic inclusions [28]. In addition, FUS is the major component of nuclear polyQ aggregates of Huntington disease (HD) as well as spinocerebellar ataxia type 1, 2, 3, and dentatorubral-pallidoluysian atrophy [29]. Histopathological analysis of FUS mutant cases illustrates distinctive FUS positive and TDP-43 negative inclusions and an earlier age of onset is noted in cases with basophilic and compact neuronal cytoplasmic [28]. FUS gene encodes for a DNA/RNA binding protein that has multiple domains and the domain at N-terminus plays a role in transcriptional activation of the gene [30,31]. As a nuclear protein and mutations at the C-terminus disrupt the transport of FUS into the nucleus, which lead to cytoplasmic localization of FUS and formation of stress granules [30,31]. Overexpression of mutant FUS in a transgenic mouse model develops progressive paralysis due to motor axonal degeneration and neuronal loss in the cortex and hippocampus [32]. In transgenic Drosophila model, it has been observed age-dependent progressive motor neuron damage when Wt, R524S or P525L mutant FUS are over-expressed in photoreceptors [33]. In addition, over-expressing WT or mutant FUS C. elegant models and knocking down endogenous FUS Zebrafish models are both used to study the mechanism of FUS mediated motor neurodegeneration [27].

#### ALS8/ vesicle associated membrane protein associated protein B (VAPB)

ALS8 is first described in a large Brazilian kindred with 28 affected male and female family members from across 4 generations [34]. Clinical onset occurs between ages 31 and 45 years, and the patients present with postural tremor, fasciculations, slow progressive upper and lower limb weakness [34]. The disease has an unusually long course compared to typical ALS. Linkage analysis reveals a novel locus at chromosome 20q13.3 and the mutation analysis depicts a mutation in the VAPB protein, which replaces the amino acid proline with the amino acid serine at position 56 [34]. A different mutation (T46I) is detected within the same domain of VAPB protein in a UK patient [35]. VAPB is an integral endoplasmic reticulum membrane protein, which has various functions such as the intracellular vesicle trafficking, lipid transport and the unfolded protein response [34,35]. Both the mutations in the VAPB domain lead to VAPB aggregation into immobile ER clusters which causes lower level of VAPB, resulting in a decreased ER anchoring of lipid-binding proteins and motor neuron degeneration [34,35]. The expression of VAPB is significantly decreased in human ALS patients and SOD1-ALS-transgenic mice, suggesting that VAPB may be involved in the pathogenesis of sALS and SOD1-linked ALS [36]. A study on mutant VAPB transgenic mice shows TDP-43 cytoplasmic inclusions, implying a link between VAPB^P56S^ mutation and TDP-43 mislocalization [37]. The VAPB^P56S^ mutation alters the binding of VAPB to tyrosine phosphatase-interacting protein 51 (PTPIP51) and increases Ca^2+^ uptake by mitochondria following the release from ER stores [38]. However, a study from Qiu et al. shows that overexpression of VAPB^P56S^ mutant in mouse spinal cord can produce abundant VAPB aggregates, but it is not associated with motor neuron degeneration, indicating that the mutant VAPB aggregates may cause motor neuron degeneration by loss of function rather than gain-of-toxicity [39].

#### ALS9/angiogenin (ANG)

ALS caused by ANG gene mutation is named as ALS9, an autosomal dominant adult onset disease presenting with the classic signs of ALS. A few patients also presented with parkinsonism and signs of FTD [40]. ANG is located at chromosome 14q11.2 and is first identified in ALS patients from Ireland and Scotland [40]. Seven missense mutations in ANG have been detected in 15 patients on a screening analysis of a large cohort of Irish, Scotish, English, Swedish and North American ALS cases, out of which 4 are fALS and the rest sALS [40]. ANG mutations are not associated with fALS in Italian population [41]. Few fALS cases are identified to have concomitant ANG mutations with FUS mutations [42] or with SOD1 mutations [43].

The ANG protein belongs to pancreatic ribonuclease superfamily, and it plays a role in inhibiting of protein translation by cleaving tRNA and helps in rRNA biogenesis and cellular proliferation [40,42,43]. ANG mediates neovascularization and promotes neurite outgrowth during early embryonic development [44]. Mutations in ANG gene cause loss of ribonucleolytic activity and nuclear translocation activity [44].

#### ALS10/ TAR DNA binding protein (TARDBP)

Mutations of gene TARDBP was first reported in fALS cases in 2008 [45]. Since then, over 40 mutations have been identified in various ethnic groups with an incidence of ~4-5% in fALS and up to 2% in sALS. TARDBP-related ALS patients present as adult-onset, autosomal dominant form of ALS with predominant limb onset and a wide variation in the age of onset (30–77 yrs) and disease duration. TARDBP mutations are observed in both ALS-FTD and FTD cases. Ubiquitinated TARDBP (TDP-43) is one of the major components of cytoplasmic inclusions in ALS and FTD [45]. TDP-43 positive cytoplasmic inclusions are found in many neurodegenerative disorders such as sALS, FTD, HD, Alzheimer’s disease (AD) and PD [46]. TDP-43 is a DNA/RNA binding protein, belonging to ribonucleoprotein family. TDP-43 is involved in a variety of functions in nucleus process, including gene transcription, RNA splicing, microRNA processing and stabilization as well as transport of mRNA [47]. Almost all the TARDBP mutations identified in ALS patients are missense mutations within the glycine-rich C-terminal region, involved in protein-protein interactions. TDP-43 has many binding targets, including FUS, vasolin containing protein (VCP), progranulin, and other transcripts encoding neurodegenerative disease-associated proteins as well as many other RNA processing genes [47]. Furthermore, by using individual-nucleotide resolution UV-cross linking and immunoprecipitation, Tollervey et al. found that TDP-43 can bind to nuclear paraspeckle assembly transcript 1 (NEAT1) and metastasis associated lung adenocarcinoma transcript 1 (MALAT1) non-coding RNAs [48,49]. It can also influence alternative splicing in position-dependent manner to Nova proteins, indicating that TDP-43 may play an essential role in splicing regulation in central nerve system [48,49]. TDP-43 interacts with mutant, but not wild-type SOD1 mRNA, thereby linking the two distinct genetic pathogenic mechanisms [50]. It is not clear how TARDBP mutations cause motor neuron degeneration, due to a loss of nuclear function or a gain of toxic function. Up to date, there are numerous animal models of TDP-43. These models expressing human wild-type or the ALS-linked mutants A315T or M337V TDP-43 (hTDP-43) under the control of the murine prion protein promoter (moPrP) display motor neuron degeneration, indicating gain-of-function mechanism [51,52]. Furthermore, the mice with high-expressing homozygous TAR4/4 display a spastic paralysis of UMN degeneration [51,52]. Up-regulation of TDP-43 in motor neurons may alter RNA metabolism via alternative splicing and RNA stability, which may increase ALS risk [51-54]. Increase of the TDP-43 level is associated with formation of TDP-43 inclusions in the nucleus and mislocalization of SMN–GEMs (Survival Motor Neuron containing Gemini of coiled bodies) and alter RNA metabolism [51,53]. On the other hand, loss of function may also play a role. For example, the lack of TDP-43 in forebrains of mice may lead to age-dependent brain atrophy. It is known that loss of TDP-43 may down-regulate Tbc1d1 protein in skeletal muscle and then induce hypermetabolism which can compromise neuronal function [51,53]. Interestingly, TARDBP knockout mice model shows decreased level of TDP-43 and body weight reduction which may result from increasing fat oxidation and accelerating fat loss in adipocytes [51].

#### ALS11/FIG 4

It is an autosomal dominant adult onset ALS with a rapid progressive course, early bulbar involvement and slight cognitive impairment [2]. Mutations in FIG 4 also known as Sac1 domain containing protein 3 (SAC3) gene located on chromosome 6q21 has been found as a causative gene for ALS11 [2]. In a screening test conducted for FIG 4 mutations in a large North European cohort of fALS and sALS patients, 5 heterozygous mutations, 1 missense, 2 splicing, and 2 truncating mutations have been identified [55].

FIG 4 is a phosphoinositide 5-phosphatase that regulates PI(3,5)P2, a signaling lipid that helps in retrograde trafficking of endosomal vesicles to the trans*-*Golgi network [56]. Mutations in FIG 4 result in neurodegeneration in sensory and autonomic ganglia, motor cortex, and striatum [55-57]. It is still not certain yet whether mutations of FIG 4 cause neurodegeneration through a dominant negative mechanism or a partial loss of function as in Charcot-Marie Tooth disease type 4 J (CMT4J) [55]. Furthermore, mutant mice with absent Vac14, a gene coding for an FIG 4 interactor, also shows neurodegeneration. Mutations in the two components of the PI(3,5)P_2_ regulatory complex, FIG 4 and Vac14, lead to cytoplasmic inclusion body formation containing p62, LC3-II and LAMP-2 in the brain, suggesting that autophagy may play a role in the gene mutations induced neurodegeneration [57].

#### ALS12/optineurin (OPTN)

ALS12 is an adult onset autosomal recessive or autosomal dominant ALS characterized by lower limb onset with UMN involvement and a slow disease progression [2]. It maps to chromosome 10p13, a genetic analysis of this gene reveals a homozygous deletion of exon 5 and another homozygous nonsense (Q398X) mutation [2]. Two homozygous mutations and a heterozygous missense mutation (E478G) are identified in a large cohort of fALS and sALS [4]. OPTN is co-localized with FUS, TDP43 and SOD1 in inclusion bodies of sALS and fALS [4]. In HD cortex, OPTN is seen in neuronal intranuclear inclusions co-labeled with ubiquitin [4]. This protein participates in multitasking cytosolic protein process involved in protein trafficking, maintenance of the Golgi complex, and exocytosis [4]. ALS12 is inherited in an autosomal recessive manner, and the OPTN protein causes neurotoxicity through the loss of function mechanism [4,5,58,59]. However, in heterozygous mutations with autosomal dominant inheritance, dominant negative effect may play a role [4,5,58,59]. Nuclear factor-κB (NF-κB), which is activated by tumor necrosis factor-alpha can be regulated by OPTN and mutations in the gene can lead to uninterrupted NF-κB neurotoxicity [4]. OPTN can be phosphorylated by protein kinase TANK binding kinase 1 (TBK1) and then promote selective autophagy function of ubiquitin-coated substance [59]. Furthermore, Koraj et al. found that OPTN can activate autophagy-lysosome pathway and clearance of protein aggregation via ubiquitin-independent manner [60].These two studies highlight that OPTN is an important factor in autophagy process.

#### ALS14/ valosin containing protein (VCP)

ALS 14 is an adult onset autosomal dominant inherited motor neuropathy with an average disease onset of 49 yrs [2]. The clinical character includes limb-onset motor neuron symptoms with rapidly progress [2]. In an Italian family with autosomal dominant ALS patients, using exome sequencing technique, a single heterozygous missense mutation in the gene coding for VCP is located on chromosome 9p13.3 [61]. Additional screening conducted on the VCP gene in 210 fALS cases and 78 autopsy-proven ALS cases has identified 3 more VCP mutations in 4 patients [61]. VCP mutations are a rare cause of fALS and clinical features could include FTD, Paget’s disease, inclusion body myopathy, and parkinsonism.

VCP is a hexameric type II ATPase of the AAA family involved in multiple cellular functions, including protein homeostasis through *e*ndoplasmic *r*eticulum-*a*ssociated *d*egradation (ERAD), Golgi biogenesis, assembly of peroxisomes, vesicle transport and fusion and autophagy [2,61]. VCP plays a role in ER stress by activating the unfolded protein response (UPR) that leads to aggregation of misfolded proteins and causes apoptotic cell death [2,61]. VCP also plays a major role in ubiquitin-dependent protein degradation [62]. Quantitative immunohistochemical study of VCP in the skin from patients with ALS and controls reveals that the proportion of VCP-positive cells in the epidermis in ALS is higher than that in controls and this proportion is higher in ALS patients with longer duration of illness [63].

#### ALS15/ALSX/ubiquilin 2 (UBQLN2)

ALSX is an adult or juvenile onset, X-linked dominantly inherited disease [2]. Patients present with UMN and LMN involvement, and UMN signs typically precede LMN signs [2]. A few of them even have dementia. UBQLN2 gene maps to chromosome Xp11. Linkage analysis in a 5 generation kindred with 19 affected candidates reveals a distinct point mutation in the coding region of UBQLN2 gene which substituted proline with histidine [64]. Later on 4 more missense mutations are identified in 4 unrelated families, all substituting proline with some other amino acid [65]. UBQLN2 is a member of the ubiquitin like protein family, which regulates the ubiquitin proteasome system (UPS) of protein degradation by delivering ubiquitinated proteins to proteasome [64-66]. Mutations in UBQLN2 impairs protein degradation pathway which can lead to an abnormal protein aggregation and neurodegeneration. UBQLN2 inclusions can be seen in the spinal cord in patients with UBQLN2 mutations [64-66]. These inclusions are also positive for other ALS proteins such as Ubiquitin, p62, TDP-43, FUS and OPTN, but negative for SOD1 [64-66]. UBQLN2 inclusions are also found in fALS, sALS and ALS-dementia cases without UBQLN2 mutations [66].

#### ALS16/SIGMAR1

ALS 16 is reported in a Saudi Arabian family [67]. Patients show UMN signs of spasticity and hyperreflexia in the initial years of life, develop LMN signs later, and progress to paralysis [67]. By homozygosity mapping, a linkage is found on chromosome 9p13.2-21.3, and using candidate gene sequencing, a mutation is identified in SIGMAR1 (SIGMA Non Opiod Intracellular Receptor1) gene [67]. Linkage studies done in 2 pedigrees, a Dutch and a Scandinavian family established a connection between familial ALS with FTD to chromosome 9p13.2-21.3 [68]. In the Scandinavian family, ALS and FTD occur separately; however, in Dutch family all the members show both ALS and FTD symptoms [67-69]. Mutation screening in 34 genes on chromosome 9p using linkage analysis in a multi-generation kindred with ALS + FTD has identified a nucleotide substitution in the 3’UTR of the SIGMAR1 gene [69]. The **SIGMAR1** protein functions as a subunit of the ligand regulated potassium channel, which can bind to neurosteroids, psychostimulants, and dextrobenzomorphans [69]. The 3’UTR alterations of the SIGMAR1 are hypothesized to alter the stability of the transcript and dysregulate the channel activity [69].

#### ALS-FTD1 and ALS-FTD2

ALS-FTD1 is an adult onset, autosomal dominant disorder which presents with the symptoms of both fALS and FTD [2,58]. ALS-FTD1 is linked to chromosome 9q21-q22 in a linkage analysis conducted on 16 ALS-FTD pedigrees [70].

Patients with ALS-FTD2 present with an adult onset, autosomal dominant fALS and FTD [2,71]. Linkage of ALS-FTD2 to chromosome 9p21 has been reported and a hexanucleotide GGGGCC repeat expansions in the chromosome 9 open reading frame 72 (C9ORF72) gene has recently been identified as the causal genetic defect of ALS-FTD2 [71]. C9ORF72 repeat expansions are currently the most frequently genetic cause of fALS and FTD, accounting for approximately 34.2 and 25.9% of the cases, respectively [72]. C9ORF72 ALS has both p62/ubiquitin- and TDP-43 positive inclusions. The loss-of function and gain-of function have been proposed. For clinical phenotype, C9ORF72 mutation manifests FTD and ALS as well as other features including memory loss, psychosis, akinetic-rigid and cerebellar signs [73]. Recent studies indicate that C9ORF72 hexanucleotide repeat could generate insoluble polypeptides specific to C9RANT immunoreactivity by non-ATG (RAN)–initiated translation, which cannot be found in other neurodegenerative diseases such as CAG repeat disorders [74,75]. These findings may provide evidence for better understanding of ALS mechanisms and treatment strategies targeting to non-ATG (RAN)-translated peptides [74-76].

#### Dynactin (DCTN1)

In a study made on large kindred with a slow progressive autosomal dominant distal hereditary motor neuronopathy, a mutation has been identified in the p150 subunit of DCTN1 gene mapped on chromosome 2p13. Later on 3 more mutations have been identified in DCTN1 gene in sALS, fALS and ALS-FTD families [77]. Dynein is a microtubule motor protein that couples ATP hydrolysis for cellular motility in cilia and flagella [78]. The binding of dynactin to dynein is critical in the axonal transport of vesicles and organelles [78]. Thus DCTN1 mutations are thought to cause neurodegeneration by impairing axonal transport in motor neurons. Additionally, G59S missense mutation in microtubule-binding domain of p150^glued^, a major component of dynein/dynactin complex can cause motor neuron degeneration [79]. In G59S p150^glued^ knock-in mice, MND-like phenotypes appear after 10 months old. Loss of spinal motor neurons, increase of reactive astrogliosis and excessive accumulation of cytoskeleton and synaptic vesicle proteins at the neuromuscular junctions are observed [79]. The motor neuron degeneration is also observed in mutant human dynactin p150^Glued^ overexpression mice model [80]. These results indicated the role of dynactin in ALS pathogenesis [80,81].

#### Other rare occurring mutant genes in fALS

Using whole genome linkage analysis with microsatellite markers in a set of families with proven ALS cases, a missense mutation in the D-amino acid oxidase (DAO) gene located on chromosome 12q22-23 has been reported in a single three generation pedigree [82]. The patients with the mutation show classical ALS signs with a rapid progression and an early bulbar involvement with a limited decline in cognitive skills [82]. DAO gene encodes a peroxisomal flavin adenine dinucleotide (FAD)-dependent oxidase that is seen in neurons and glial cells of brainstem and spinal cord [83]. R199W mutations in DAO decrease the cell viability, increase the formation of ubiquitinated aggregates and enhance the apoptosis in primary motor neuron cultures [82,83]. When motor neurons are cultured alongside the astrocytes expressing R199W mutation, similar features are documented, indicating that motor neuron death induced by this mutation can be mediated by both cell autonomous and non-cell autonomous processes [82]. However it is still not clear if the neurodegenerative effect is from the accumulation of the aberrant proteins or from an impaired enzyme activity [83,84]. Impaired enzyme activity leads to the buildup of D-amino acids like D-serine, an agonist at the glycine site of the N-methyl-DL-aspartic acid (NMDA) receptor, enhancing glutamate transmission and may exacerbate motor neuron death [84]. D-serine is increased both in ALS patients and in SOD1^G93A^ mouse model of ALS [84].

ALS3 has been mapped to chromosome 18q21 in a large European family [2]. This locus consists of almost 50 genes and the exact gene causing the disease is not yet to be identified. In one of the pedigrees, a genetic subtype of ALS, designated ALS7, is linked to chromosome 20ptel-p13 [2]. The ALS patients in the family show the signs of adult onset fALS with rapid disease progression [2].

### Genetics of sALS

The incidence of sALS is more in men than in women [1,2]. The only clinical feature that distinguishes recognized sporadic from apparently hereditary ALS is older age of onset in the former [1-3]. All the clinical features reported in hereditary cases including the extrapyramidal and cerebellar signs or cognitive involvement have also been observed in sALS [1-3]. The cause of sALS in most cases is not known. Several fALS genes such as C9ORF72, TDP-43, FUS and SOD1 have also been reported in a small proportion of sALS cases [5]. The crosslink between genetic and environmental factors may contribute to the pathogenesis of sALS [1-4]; Table 2. Discovering causative mechanisms in sALS will facilitate effective treatments and cures for this disease.

**Table 2:** The genetics Of sALS

**Gene**	**Protein**	**Chromosomal locus**	**Variant associated with ALS**
APEX1	Apurinic Endonuclease DNA repair enzyme 1	14q11.2	SNP associations
ATXN2	Ataxin-2	12q24.12	Poly Q repeats
CHMP2B	Chromatin Modifying Protein 2B	3p11.2	Mutations
HFE	Haemochromatosis	6p22.2	SNP associations
NEFH	Neuro filament Heavy	22q12.2	Deletion and Insertions
SMN1	Survival Motor Neuron 1	5q12.2-q13.3	Abnormal copy number of genes
SMN2	Survival Motor Neuron 2	5q12.2-q13.3	Abnormal copy number of genes
PON 1,2,3	Paraoxonase	7q21.3	SNP associations and mutations
PRPH	Peripherin	12q13.12	mutations
VEGF	Vascular Endothelial Growth Factor	6p21	Promoter SNP’s
PGRN	Progranulin	17q21.31	Deletions

Although the cause of sALS is not known, the crosslink between genetic and environmental factors may contribute to the pathogenesis of sALS. APEX1, ATXN2, CHMP2B, HFE, NEFH, SMN1, SMN2, PON 1, PON2, PON3, PRPH, VEGF and PGRN are some of the genes associated with sALS. Single Nucleotide Polymorphisms (SNP’s), Polyglutamine (Poly Q) repeats, abnormal copy number of genes, deletion and insertion mutations are some of the associations found with the genes known to cause sporadic ALS.

Abbreviations for Table 2: *APEX1* Apurinic Endonuclease DNA repair enzyme 1, *ATXN2* Ataxin-2, *CHMP2B* Charged multivesicular body protein 2B, *HFE* Haemochromatosis, *NEFH* Neuro filament Heavy, *SMN* Survival Motor Neuron, *PON* Paraoxonase, *PRPH* Peripherin, *VEGF* Vascular Endothelial Growth Factor, *PGRN* Progranulin.

#### Apurinic endonuclease (APEX1)

Mutation analysis conducted on 117 Scottish sALS patients shows a SNP association ending in a D148E amino-acid change in APEX1 [85]. However, later on two other studies fail to confirm this finding [86]. This might imply that APEX1 mutations may cause sALS in a particular geographic population. APEX1 participates in the process of DNA repair and DNA binding of transcription factors and plays a protective role against oxidative stress [87]. APEX1 is a unique redox factor, but the mutants will lose redox activity and fail to stimulate cell proliferation. APEX1’s redox function is also shown to be neuroprotective after exposure to ionizing radiation which produces reactive oxygen species and oxidative DNA damage in neurons [87].

#### Charged multivesicular body protein 2B (CHMP2B)

A splice site mutation of CHMP2B is first revealed in a Danish family as a rare cause of FTD accounting for <1% of the total cases [88]. Patients present with predominant LMN phenotype and one of the patients even shows signs of FTD [88,89]. Later on CHMP2B gene sequencing in 433 ALS patients from England has identified three missense mutations in fALS and sALS cases [88]. CHMP2B belongs to CHMP family and this protein is components of ESCRT-III, a complex involved in degradation of surface receptor proteins and in the trafficking of proteins between plasma membrane, trans-golgi network and lysosomes [88,89]. CHMP2B mutations in FTD cases can result in disrupted endosomal structure which is similar to those seen in alsin mutations [88,89]. In addition, CHMP2B mutations lead to dendritic retraction and autophagosomal aggregation in cortical neurons and in hippocampal neurons, implying that CHMP2B is needed for dendritic spine growth and maturation [90,91].

#### Neurofilaments

Neurofilaments are neuronal cytoplasmic intermediate filaments that form the cytoskeleton of myelinated axons. Neurofilaments are formed by the subunits of different molecular masses light (NEFL), medium (NEFM), and heavy (NEFH), which are encoded by different genes [92]. The abnormal accumulation of neurofilaments in the cell bodies and proximal axons of motor neurons is pathognomonic [92]. Association between NEFH and ALS has been shown in a small group of patients [92]. Overexpression of NEFH, NEFL, and peripherin, as well as the decreased activity of the dynein protein can cause paralytic symptoms in mice associated with axonal atrophy and motor dysfunction [93]. NEFL is required for neurofilament assembly; thus, in NEFL null mice, NEFM and NEFH can not assemble and transport accurately, leading to reduced levels in axons [93]. Mutations in NEFL are known to cause a form of hereditary sensory and motor neuropathy, Charcot-Marie-Tooth disease [94]. The C-terminal of NEFH contains phosphorylation motifs called lys-ser-pro repeats that have 2 polymorphic variants of short and long repeats. Homozygosity for the short repeat allele is associated with sALS in a study conducted on Russian patients [95]. Deletions and insertions in the C-terminal KSP repeats of NEFH are noted in some sALS patients [92].

#### Paraoxonase

The paraoxonases (PON) have 3 units (PON1, PON2, and PON3) located on 80-kb block of chromosome 7q21.3. PON1 and PON3 are primarily expressed in liver and reach to the blood where they are associated with high-density lipoproteins and may protect against atherosclerosis, whereas PON2 is expressed in many tissues [96]. PON1 and PON2 have been found to express in mouse brain [96,97]. PON proteins are involved in the hydrolysis of lactones and in the detoxification of organophosphate pesticides, neurotoxins and aromatic esters. The PON gene polymorphisms are found to be associated with sALS [98,99]. The haploblock of high linkage disequilibrium spanning PON2 and PON3 is also associated with sALS [98]. Recently a genomic DNA sequencing study has identified seven mutations in the PON genes in patients with fALS and sALS [100]. Furthermore, it has been shown that neurotoxicity caused by oxidative stress in PON2 knockout mice is more significant than in wild type mice, indicating that PON2 might have a neuroprotective effect against oxidative stress [96].

#### Peripherin (PRPH)

PRPH is a type III intermediate filament similar to neurofilaments. It acts as a cytoskeletal protein and is present in the neurons of the peripheral nervous system [101]. PRPH expression is increased in the spinal motor neurons after neuronal injury, indicating its role in axonal regeneration [101]. Lewy body-like ubiquitinated inclusions and Bunina bodies that are seen in some ALS patients contain PRPH [101]. Overexpression of wild-type PRPH in transgenic mice develops a selective, large scale late-onset motor neuron degeneration characterized by intermediate filament inclusions [102]. Later on 2 homozygous missense mutations have been identified in PRPH gene [103]. A study on 122 Italian ALS patients has identified eighteen sequence variations on PRPH including 2 missense variations, namely p.R133P and p.D141Y [103]. These two variants are predicted to have a deleterious effect on protein structure or function [103]. PRPH splice variants are noted in certain mouse models which may contribute to the ALS pathogenesis. A pathogenic isoform of PRPH (Per61) is found in motor neurons of mutant SOD1 mice but not in wild-type transgenic mice [104]. Per61 is not able to assemble properly, but it can form intracellular aggregates and then cause neurotoxicity [104]. Per61 is also observed in the motor neurons from mutant TDP-43 transgenic mice but not from wild-type TDP-43 mice [105]. Recently, another PRPH splice variant (Per28) is found in ALS patients, overexpression of which leads to PRPH aggregation in transgenic mouse model [106].

#### Survival motor neuron (SMN) 1 AND 2

SMN is in a structure named ‘GEMS’ (Gemini of the coiled bodies) which has important function in mRNA metabolism. SMN and its associated protein, SMN-interacting protein-1 (SIP1), form a complex with several spliceosomal snRNP (small nuclear Ribo Nucleo Protein) [107,108]. The impaired assembly and function of the spliceosome could cause motor neuron degeneration. Homozygous deletion mutations of SMN1 gene on chromosome 5 cause the autosomal recessive disorder spinal muscular atrophy (SMA), a fatal childhood-onset neuromuscular disease characterized by the degeneration of spinal cord motor neurons and subsequent muscle paralysis [107]. A study on 110 ALS patients and 100 controls found an increased frequency of SMN2 deletions in sALS patients (16%) in controls (4%), suggesting that SMN2 deletion may be a risk factor for ALS [108]. In another study on 167 ALS patients for SMN1 and SMN2 copy number, 16% ALS patients have an abnormal copy numbers of SMN1 [109]. Although the involved SMN gene is different in the two studies, both suggest that the SMN copy numbers is associated with ALS. Homozygous deletion mutations in SMN genes are not found in ALS but an abnormal copy numbers in SMN1 could increase the risk for ALS [109]. Further studies are needed to clarify the role of the SMN1 and SMN2 genes in sALS.

#### Vascular endothelial growth factor (VEGF)

VEGF plays a role in angiogenesis in response to hypoxia. Deletion of hypoxia-responsive element from the VEGF promoter in mice can cause the late-onset motor neuron degeneration similar to ALS [110]. Two experiments conducted on mutant SOD1 transgenic mice by overexpressing VEGF, one through delivery in muscle and the other through intra-cerebroventricular administration, prolong the survival in mutant SOD1 rats [111]. Spinal cords of ALS patients show reduced expression of VEGF and its receptor [112]. All these lead to the identification of VEGF as a possible candidate gene for ALS. Mutation sequencing of the VEGF gene and VEGF promotor in ALS patients fails to identify any specific mutations. However, a study performed on 750 ALS patients and over 1200 controls from Sweden, Belgium and England reveal that certain SNPs in the VEGF gene are associated with the lower level of VEGF expression and higher risk of ALS, suggesting a link between VEGF levels and ALS susceptibility [113].

#### Progranulin (PGRN)

It is a glycoprotein that is a precursor for granulins or epithelins involved in the development, inflammation and wound repair [114]. PGRN has also been linked to tumorigenesis and activated microglia in several neurodegenerative diseases [114]. Nonsense and deletion mutations of PGRN are known to be the cause of ubiquitin positive, tau negative FTD (FTDU) [115]. To date, only a single study links PGRN mutations to ALS [115].

#### Ataxin-2 (ATXN2)

Ataxin-2, the protein encoded by the ATXN2 gene, contains a polyglutamine (polyQ) tract with normally 22–23 repeats. Intermediate length polyQ repeats (23–34) are found in ALS patients [97]. ATXN2 and TDP-43 form a RNA-dependent complex, and longer polyQ repeats stabilize ATXN2 and increases its interaction with TDP-43. This leads to enhanced dislocation of TDP-43 into the cytoplasm in the spinal cord motor neurons in ALS patients [116]. ATXN2 is also able to interact with endophilins involved in synaptic vesicle endocytosis [117].

## Conclusion

There is a great heterogeneity for genetic spectrum of fALS and sALS. Several genes in ALS are known to cause many other neurodegenerative diseases, such as alsin with PLS and infantile onset ascending hereditary spastic paralysis (IAHSP), Senataxin with SCAR1 or AOA2, spatacsin with HSP, VAPB with SMA, FIG 4 with CMT type 4 J, OPTN with primary open angle glaucoma. In addition, there is a clinical and pathological overlap between ALS and FTLD. A number of autosomal-dominant genes have been described as primarily cause ALS or FTD such as VCP, and TARDBP. The presence of two neurodegenerative phenotypes within the same family and even within the same individual naturally raises question about the genetic and environmental interaction on the disease initiation.

Using linkage analysis, candidate gene studies and genome wide association studies, about 1/3 fALS and a small number of sALS have been revealed the disease-caused genes. However, despite all the progress achieved, the causes of large majority of sALS remain unknown. With the advanced genetics technology, we can expect that the number of genes involved in fALS as well as sALS will continue to increase. In addition, the utilization of transgenic animal models may provide useful tool to study the pathogenesis of ALS. Emphasis should also be made on elaborating the gene-environment interactions and crosslink in ALS, as 90% of the cases are sporadic in origin, which may help better understand the nature of the disease.

It is difficult to predict the future outcome in ALS research, but the identification of novel genes, gene modifiers and the different molecular pathways caused by the aberrant genes, might advance our research in this area. Hopefully, the use of the deep sequencing techniques, transgenic animal models, retrograde studies on available data and prospective design of future studies, may help broaden our vision in understanding the ALS genetics and pathogenesis. Strategic approach based on new ALS genes and drug trials on animal models should enable us to uncover new treatment modalities.

## Abbreviations

A’D: Alzheimer’s disease; AD: Autosomal dominant; ALS: Amyotrophic lateral sclerosis; ANG: Angiogenin; AOA2: Ataxia Ocular Apraxia 2; APEX1: Apurinic Endonuclease DNA repair enzyme 1; AR: Autosomal recessive.ATXN2, Ataxin-2; C9ORF72: Chromosome 9 open reading frame 72; CHMP2B: Chromatin Modifying Protein 2B; CMT 4 J: Charcot-Marie Tooth disease type 4 J; DAO: D-Amino Acid Oxidase; DCTN: Dynactin; ESCRT-III: endosomal sorting complex required for transport III; fALS: familial ALS; FIG 4: Factor Induced Gene 4; FTD: Frontal-temporal dementia; GEF: Guanine nucleotide exchange factor; HD: Huntington’s disease; HFE: Haemochromatosis; HSP: Hereditary spastic paraplegia; IAHSP: Infantile onset ascending hereditary spastic paralysis; IBMPFD: Inclusion body myopathy with Pagets disease and fronto- temporal dementia; IGHMBP2: Immunoglobulin mu binding protein 2 gene; LMN: Lower motor neurons; NEFH: Neuro filament heavy; NF-κB: Nuclear factor-κB; OPTN: Optineurin; PD: Parkinson disease; PGRN: Progranulin; PLS: Primary lateral sclerosis; PON: Paraoxonase; PRPH: Peripherin; sALSM: sporadic ALS; SCAR 1: Autosomal Recessive Spino-cerebellar ataxia; SETX: Senataxin; SIGMAR1: Sigma Non Opiod Intracellular Receptor; SIP1: SMN-interacting protein-1; SMA: Spinal Muscular Atrophy; SMN1: Survival Motor Neuron 1; SMN2: Survival Motor Neuron 2; SOD1: Superoxide Dismutase 1; SPG: spatacsin; UBQLN2: Ubiquilin 2 UMN, upper motor neurons; UPS: Ubiquitin–proteasome system; VAPB: Vesicle associated membrane protein associated protein B; VCP: Valosin containing protein; VEGF: Vascular endothelial growth factor.

## Competing interests

The authors declare that they have no competing interests.

## Authors’ contribution

SC contributed to update genetic and mechanism of ALS most part of manuscript, make revision and correction. PS contributed to the draft of the paper and figure. XZ contributed to the revision of the paper. All the authors have read and approved the final manuscript.

## References

[B1] Appel SH Zhao W Beers DR Henkel JS The microglial-motoneuron dialogue in ALS Acta Myol 2011 30 4 8 21842586 PMC3185827

[B2] Pasinelli P Brown RH Molecular biology of amyotrophic lateral sclerosis: insights from genetics Nat Rev Neurosci 2006 7 710 723 16924260 10.1038/nrn1971

[B3] Czaplinski A Yen AA Simpson EP Appel SH Slower disease progression and prolonged survival in contemporary patients with amyotrophic lateral sclerosis: is the natural history of amyotrophic lateral sclerosis changing? Arch Neurol 2006 63 1139 1143 10.1001/archneur.63.8.1139 16908741

[B4] Maruyama H Morino H Ito H Izumi Y Kato H Watanabe Y Kinoshita Y Kamada M Nodera H Suzuki H Komure O Matsuura S Kobatake K Morimoto N Abe K Suzuki N Aoki M Kawata A Hirai T Kato T Ogasawara K Hirano A Takumi T Kusaka H Hagiwara K Kaji R Kawakami H Mutations of optineurin in amyotrophic lateral sclerosis Nature 2010 465 223 226 10.1038/nature08971 20428114

[B5] Turner MR Hardiman O Benatar M Brooks BR Chio A de Carvalho M Ince PG Lin C Miller RG Mitsumoto H Nicholson G Ravits J Shaw PJ Swash M Talbot K Traynor BJ Van den Berg LH Veldink JH Vucic S Kiernan MC Controversies and priorities in amyotrophic lateral sclerosis Lancet Neurol 2013 12 310 322 10.1016/S1474-4422(13)70036-X 23415570 PMC4565161

[B6] Rezania K Yan J Dellefave L Deng HX Siddique N Pascuzzi RT Siddique T Roos RP A rare Cu/Zn superoxide dismutase mutation causing familial amyotrophic lateral sclerosis with variable age of onset, incomplete penetrance and a sensory neuropathy Amyotroph Lateral Scler Other Motor Neuron Disord 2003 4 162 166 10.1080/aml.4.3.162.166 13129803

[B7] Lopate G Baloh RH Al-Lozi MT Miller TM Fernandes Filho JA Ni O Leston A Florence J Schierbecker J Allred P Familial ALS with extreme phenotypic variability due to the I113T SOD1 mutation Amyotroph Lateral Scler 2010 11 232 236 10.3109/17482960902898069 20184521

[B8] Mackenzie IR Bigio EH Ince PG Geser F Neumann M Cairns NJ Kwong LK Forman MS Ravits J Stewart H Eisen A McClusky L Kretzschmar HA Monoranu CM Highley JR Kirby J Siddique T Shaw PJ Lee VM Trojanowski JQ Pathological TDP-43 distinguishes sporadic amyotrophic lateral sclerosis from amyotrophic lateral sclerosis with SOD1 mutations Ann Neurol 2007 61 427 434 10.1002/ana.21147 17469116

[B9] Gurney ME The use of transgenic mouse models of amyotrophic lateral sclerosis in preclinical drug studies J Neurol Sci 1997 152 Suppl 1 S67 S73 9419057 10.1016/s0022-510x(97)00247-5

[B10] Massilamany C Gangaplara A Kim H Stanford C Rathnaiah G Steffen D Lee J Reddy J Copper-zinc superoxide dismutase-deficient mice show increased susceptibility to experimental autoimmune encephalomyelitis induced with myelin oligodendrocyte glycoprotein 35–55 J Neuroimmunol 2013 256 19 27 10.1016/j.jneuroim.2012.12.004 23294897 PMC4100484

[B11] Hadano S Hand CK Osuga H Yanagisawa Y Otomo A Devon RS Miyamoto N Showguchi-Miyata J Okada Y Singaraja R Figlewicz DA Kwiatkowski T Hosler BA Sagie T Skaug J Nasir J Brown RH Jr Scherer SW Rouleau GA Hayden MR Ikeda JE A gene encoding a putative GTPase regulator is mutated in familial amyotrophic lateral sclerosis 2 Nature Genet 2001 29 166 173 10.1038/ng1001-166 11586298

[B12] Kanekura K Hashimoto Y Niikura T Aiso S Matsuoka M Nishimoto I Alsin, the product of ALS2 gene, suppresses SOD1 mutant neurotoxicity through RhoGEF domain by interacting with SOD1 mutants J Biol Chem 2004 279 19247 19256 10.1074/jbc.M313236200 14970233

[B13] Li Q Spencer NY Pantazis NJ Engelhardt JF Alsin and SOD1(G93A) proteins regulate endosomal reactive oxygen species production by glial cells and proinflammatory pathways responsible for neurotoxicity J Biol Chem 2011 286 40151 40162 10.1074/jbc.M111.279711 21937428 PMC3220533

[B14] Otomo A Kunita R Suzuki-Utsunomiya K Mizumura H Onoe K Osuga H Hadano S Ikeda JE ALS2/alsin deficiency in neurons leads to mild defects in macropinocytosis and axonal growth Biochem Biophys Res Commun 2008 370 87 92 10.1016/j.bbrc.2008.01.177 18358238

[B15] Cai H Shim H Lai C Xie C Lin X Yang WJ Chandran J ALS2/alsin knockout mice and motor neuron diseases Neurodegener Dis 2008 5 359 366 10.1159/000151295 18714162 PMC2556598

[B16] Gros-Louis F Kriz J Kabashi E McDearmid J Millecamps S Urushitani M Lin L Dion P Zhu Q Drapeau P Julien JP Rouleau GA Als2 mRNA splicing variants detected in KO mice rescue severe motor dysfunction phenotype in Als2 knock-down zebrafish Hum Mol Genet 2008 17 2691 2702 10.1093/hmg/ddn171 18558633

[B17] Chance PF Rabin BA Ryan SG Ding Y Scavina M Crain B Griffin JW Cornblath DR Linkage of the gene for an autosomal dominant form of juvenile amyotrophic lateral sclerosis to chromosome 9q34 Am J Hum Genet 1998 62 633 640 10.1086/301769 9497266 PMC1376963

[B18] Chen Y-Z Bennett CL Huynh HM Blair IP Puls I Irobi J Dierick I Abel A Kennerson ML Rabin BA Nicholson GA Auer-Grumbach M Wagner K De Jonghe P Griffin JW Fischbeck KH Timmerman V Cornblath DR Chance PF DNA/RNA helicase gene mutations in a form of juvenile amyotrophic lateral sclerosis (ALS4) Am J Hum Genet 2004 74 1128 1135 10.1086/421054 15106121 PMC1182077

[B19] Moreira M-C Klur S Watanabe M Nemeth AH Le Ber I Moniz J-C Tranchant C Aubourg P Tazir M Schöls L Pandolfo P Schulz JB Pouget J Calvas P Shizuka-Ikeda M Shoji M Tanaka M Izatt L Shaw CE M’Zahem A Dunne E Bomont P Benhassine T Bouslam N Stevanin G Brice A Guimarães J Mendonça P Barbot C Coutinho P Sequeiros J Dürr A Warter JM Koenig M Senataxin, the ortholog of a yeast RNA helicase, is mutant in ataxia-ocular apraxia 2 Nature Genet 2004 36 225 227 10.1038/ng1303 14770181

[B20] Grohmann K Schuelke M Diers A Hoffmann K Lucke B Adams C Bertini E Leonhardt-Horti H Muntoni F Ouvrier R Pfeufer A Rossi R Van Maldergem L Wilmshurst JM Wienker TF Sendtner M Rudnik-Schöneborn S Zerres K Hübner C Mutations in the gene encoding immunoglobulin mu- binding protein 2 cause spinal muscular atrophy with respiratory distress type 1 Nat Genet 2001 29 75 77 10.1038/ng703 11528396

[B21] Skourti-Stathaki K Proudfoot NJ Gromak N Human senataxin resolves RNA/DNA hybrids formed at transcriptional pause sites to promote Xrn2-dependent termination Mol Cell 2011 42 794 805 10.1016/j.molcel.2011.04.026 21700224 PMC3145960

[B22] Hentati A Ouahchi K Pericak-Vance MA Nijhawan D Ahmad A Yang Y Rimmler J Hung W Schlotter B Ahmed A Ben Hamida M Hentati F Siddique T Linkage of a commoner form of recessive amyotrophic lateral sclerosis to chromosome 15q15-q22 markers Neurogenetics 1998 2 55 60 10.1007/s100480050052 9933301

[B23] Stevanin G Santorelli FM Azzedine H Coutinho P Chomilier J Denora PS Martin E Ouvrard-Hernandez AM Tessa A Bouslam N Lossos A Charles P Loureiro JL Elleuch N Confavreux C Cruz VT Ruberg M Leguern E Grid D Tazir M Fontaine B Filla A Bertini E Durr A Brice A Mutations in SPG11, encoding spatacsin, are a major cause of spastic paraplegia with thin corpus callosum Nat Genet 2007 39 366 372 10.1038/ng1980 17322883

[B24] Orlacchio A Babalini C Borreca A Patrono C Massa R Basaran S Munhoz RP Rogaeva EA St George-Hyslop PH Bernardi G Kawarai T SPATACSIN mutations cause autosomal recessive juvenile amyotrophic lateral sclerosis Brain 2010 133 591 598 10.1093/brain/awp325 20110243 PMC2822627

[B25] Murmu RP Martin E Rastetter A Esteves T Muriel MP El Hachimi KH Denora PS Dauphin A Fernandez JC Duyckaerts C Brice A Darios F Stevanin G Cellular distribution and subcellular localization of spatacsin and spastizin, two proteins involved in hereditary spastic paraplegia Mol Cell Neurosci 2011 47 191 202 10.1016/j.mcn.2011.04.004 21545838

[B26] Sapp PC Hosler BA McKenna-Yasek D Chin W Gann A Genise H Gorenstein J Huang M Sailer W Scheffler M Valesky M Haines JL Pericak-Vance M Siddique T Horvitz HR Brown RH Jr Identification of two novel loci for dominantly inherited familial amyotrophic lateral sclerosis Am J Hum Genet 2003 73 397 403 10.1086/377158 12858291 PMC1180377

[B27] Lanson NA Jr Pandey UB FUS-related proteinopathies: lessons from animal models Brain Res 2012 1462 3 15 22342159 10.1016/j.brainres.2012.01.039

[B28] Bäumer D Hilton D Paine SM Turner MR Lowe J Talbot K Ansorge O Juvenile ALS with basophilic inclusions is a FUS proteinopathy with FUS mutations Neurology 2010 75 611 618 10.1212/WNL.0b013e3181ed9cde 20668261 PMC2931770

[B29] Doi H Koyano S Suzuki Y Nukina N Kuroiwa Y The RNA-binding protein FUS/TLS is a common aggregate-interacting protein in polyglutamine diseases Neurosci Res 2010 66 131 133 10.1016/j.neures.2009.10.004 19833157

[B30] Dormann D Rodde R Edbauer D Bentmann E Fischer I Hruscha A Than ME Mackenzie IR Capell A Schmid B Neumann M Haass C ALS-associated fused in sarcoma (FUS) mutations disrupt transportin-mediated nuclear import EMBO J 2010 29 2841 2857 10.1038/emboj.2010.143 20606625 PMC2924641

[B31] Bosco DA Lemay N Ko HK Zhou H Burke C Kwiatkowski TJ Jr Sapp P McKenna-Yasek D Brown RH Jr Hayward LJ Mutant FUS proteins that cause amyotrophic lateral sclerosis incorporate into stress granules Hum Mol Genet 2010 19 4160 4175 10.1093/hmg/ddq335 20699327 PMC2981014

[B32] Haung C Zhou H Tong J Chen H Liu YJ Wang D Wei X Xia XG FUS transgenic rats develop the phenotypes of amyotropic lateral sclerosis and frontotemporal lobar degeneration PLoS Genet 2011 7 e100 10.1371/journal.pgen.1002011 PMC3048370 21408206

[B33] Chen Y Yang M Deng J Chen X Ye Y Zhu L Liu J Ye H Shen Y Li Y Rao EJ Fushimi K Zhou X Bigio EH Mesulam M Xu Q Wu JY Expression of human FUS protein in Drosophila leads to progressive neurodegeneration Protein Cell 2011 2 477 486 10.1007/s13238-011-1065-7 21748598 PMC3563268

[B34] Nishimura AL Mitne-Neto M Silva HC Richieri-Costa A Middleton S Cascio D Kok F Oliveira JR Gillingwater T Webb J Skehel P Zatz M A mutation in the vesicle-trafficking protein VAPB causes late-onset spinal muscular atrophy and amyotrophic lateral sclerosis Am J Hum Genet 2004 75 822 831 10.1086/425287 15372378 PMC1182111

[B35] Chen HJ Anagnostou G Chai A Withers J Morris A Adhikaree J Pennetta G de Belleroche JS Characterization of the properties of a novel mutation in VAPB in familial amyotrophic lateral sclerosis J Biol Chem 2010 285 40266 40281 10.1074/jbc.M110.161398 20940299 PMC3001007

[B36] Teuling E Ahmed S Haasdijk E Demmers J Steinmetz MO Akhmanova A Jaarsma D Hoogenraad CC Motor neuron disease-associated mutant vesicle-associated membrane protein-associated protein (VAP) B recruits wild-type VAPs into endoplasmic reticulum-derived tubular aggregates J Neurosci 2007 27 9801 9815 10.1523/JNEUROSCI.2661-07.2007 17804640 PMC6672975

[B37] Tudor EL Galtrey CM Perkinton MS Lau KF De Vos KJ Mitchell JC Ackerley S Hortobágyi T Vámos E Leigh PN Klasen C McLoughlin DM Shaw CE Miller CC Amyotrophic lateral sclerosis mutant vesicle-associated membrane protein-associated protein-B transgenic mice develop TAR-DNA-binding protein-43 pathology Neuroscience 2010 167 774 785 10.1016/j.neuroscience.2010.02.035 20188146

[B38] De Vos KJ Mórotz GM Stoica R Tudor EL Lau KF Ackerley S Warley A Shaw CE Miller CC VAPB interacts with the mitochondrial protein PTPIP51 to regulate calcium homeostasis Hum Mol Genet 2012 21 1299 1311 10.1093/hmg/ddr559 22131369 PMC3284118

[B39] Qiu L Qiao T Beers M Tan W Wang H Yang B Xu Z Widespread aggregation of mutant VAPB associated with ALS does not cause motor neuron degeneration or modulate mutant SOD1 aggregation and toxicity in mice Mol Neurodegener 2013 8 1 10.1186/1750-1326-8-1 23281774 PMC3538568

[B40] Greenway MJ Alexander MD Ennis S Traynor BJ Corr B Frost E Green A Hardiman O A novel candidate region for ALS on chromosome 14q11.2 Neurology 2004 63 1936 1938 10.1212/01.WNL.0000144344.39103.F6 15557516

[B41] Corrado L Battistini S Penco S Bergamaschi L Testa L Ricci C Giannini F Greco G Patrosso MC Pileggi S Causarano R Mazzini L Momigliano-Richiardi P D’Alfonso S Variations in the coding and regulatory sequences of the angiogenin (ANG) gene are not associated to ALS (amyotrophic lateral sclerosis) in the Italian population J Neurol Sci 2007 258 123 127 10.1016/j.jns.2007.03.009 17462671

[B42] Millecamps S Salachas F Cazeneuve C Gordon P Bricka B Camuzat A Guillot-Noël L Russaouen O Bruneteau G Pradat PF Le Forestier N Vandenberghe N Danel-Brunaud V Guy N Thauvin-Robinet C Lacomblez L Couratier P Hannequin D Seilhean D Le Ber I Corcia P Camu W Brice A Rouleau G LeGuern E Meininger V SOD1, ANG, VAPB, TARDBP, and FUS mutations in familial amyotrophic lateral sclerosis: genotype-phenotype correlations J Med Genet 2010 47 554 560 10.1136/jmg.2010.077180 20577002

[B43] Luigetti M Lattante S Zollino M Conte A Marangi G Del Grande A Sabatelli M SOD1 G93D sporadic amyotrophic lateral sclerosis (SALS) patient with rapid progression and concomitant novel ANG variant Neurobiol Aging 1924 2011 32 10.1016/j.neurobiolaging.2011.04.004 21621297

[B44] Padhi AK Kumar H Vasaikar SV Jayaram B Gomes J Mechanisms of loss of functions of human angiogenin variants implicated in amyotrophic lateral sclerosis PLoS One 2012 7 e32479 10.1371/journal.pone.0032479 22384259 PMC3288110

[B45] Sreedharan J Blair IP Tripathi VB Hu X Vance C Rogelj B Ackerley S Durnall JC Williams KL Buratti E Baralle F de Belleroche J Mitchell JD Leigh PN Al-Chalabi A Miller CC Nicholson G Shaw CE TDP-43 mutations in familial and sporadic amyotrophic lateral sclerosis Science 2008 319 1668 1672 10.1126/science.1154584 18309045 PMC7116650

[B46] Da Cruz S Cleveland DW Understanding the role of TDP-43 and FUS/TLS in ALS and beyond Curr Opin Neurobiol 2011 21 904 919 10.1016/j.conb.2011.05.029 21813273 PMC3228892

[B47] Buratti E Baralle FE The multiple roles of TDP-43 in pre-mRNA processing and gene expression regulation RNA Biol 2010 7 420 429 10.4161/rna.7.4.12205 20639693

[B48] Polymenidou M Lagier-Tourenne C Hutt KR Huelga SC Moran J Liang TY Ling SC Sun E Wancewicz E Mazur C Kordasiewicz H Sedaghat Y Donohue JP Shiue L Bennett CF Yeo GW Cleveland DW Long pre-mRNA depletion and RNA missplicing contribute to neuronal vulnerability from loss of TDP-43 Nat Neurosci 2011 14 459 468 10.1038/nn.2779 21358643 PMC3094729

[B49] Tollervey JR Curk T Rogelj B Briese M Cereda M Kayikci M König J Hortobágyi T Nishimura AL Zupunski V Patani R Chandran S Rot G Zupan B Shaw CE Ule J Characterizing the RNA targets and position-dependent splicing regulation by TDP-43 Nat Neurosci 2011 14 452 458 10.1038/nn.2778 21358640 PMC3108889

[B50] Higashi S Tsuchiya Y Araki T Wada K Kabuta T TDP-43 physically interacts with amyotrophic lateral sclerosis-linked mutant CuZn superoxidedismutase Neurochem Int 2010 57 906 913 10.1016/j.neuint.2010.09.010 20933032

[B51] Tsao W Jeong YH Lin S Ling J Price DL Chiang PM Wong PC Rodent models of TDP-43: recent advances Brain Res 2012 1462 26 39 22608070 10.1016/j.brainres.2012.04.031 PMC3613131

[B52] Gendron TF Petrucelli L Rodent models of TDP-43 proteinopathy: investigating the mechanisms of TDP-43-mediated neurodegeneration J Mol Neurosci 2011 45 486 499 10.1007/s12031-011-9610-7 21811811 PMC3207125

[B53] Xu ZS Does a loss of TDP-43 function cause neurodegeneration? Mol Neurodegener 2012 7 27 10.1186/1750-1326-7-27 22697423 PMC3419078

[B54] Joyce PI Fratta P Fisher EM Acevedo-Arozena A SOD1 and TDP- 43 animal models of amyotrophic lateral sclerosis: recent advances in understanding disease toward the development of clinical treatments Mamm Genome 2011 22 420 448 10.1007/s00335-011-9339-1 21706386

[B55] Chow CY Zhang Y Dowling JJ Jin N Adamska M Shiga K Szigeti K Shy ME Li J Zhang X Lupski JR Weisman LS Meisler MH Mutation of FIG 4 causes neurodegeneration in the pale tremor mouse and patients with CMT4J Nature 2007 448 68 72 10.1038/nature05876 17572665 PMC2271033

[B56] Michell RH Dove SK A protein complex that regulates PtdIns(3,5)P2 levels EMBO J 2009 28 86 97 10.1038/emboj.2008.270 19158662 PMC2634735

[B57] Zhang Y Zolov SN Chow CY Slutsky SG Richardson SC Piper RC Yang B Nau JJ Westrick RJ Morrison SJ Meisler MH Weisman LS Loss of Vac14, a regulator of the signaling lipid phosphatidylinositol 3,5-biphosphate, results in neurodegeneration in mice Proc Natl Acad Sci USA 2007 104 17518 17523 10.1073/pnas.0702275104 17956977 PMC2077288

[B58] Sakaguchi T Irie T Kawabata R Yoshida A Maruyama H Kawakami H Optineurin with amyotrophic lateral sclerosis-related mutations abrogates inhibition of interferon regulatory factor-3 activation Neurosci Lett 2011 505 279 281 10.1016/j.neulet.2011.10.040 22040667

[B59] Wild P Farhan H McEwan DG Wagner S Rogov VV Brady NR Richter B Korac J Waidmann O Choudhary C Dötsch V Bumann D Dikic I Phosphorylation of the autophagy receptor optineurin restricts Salmonella growth Science 2011 333 228 233 10.1126/science.1205405 21617041 PMC3714538

[B60] Korac J Schaeffer V Kovacevic I Clement AM Jungblut B Behl C Terzic J Dikic I Ubiquitin-independent function of optineurin in autophagic clearance of protein aggregates J Cell Sci 2013 126 580 592 10.1242/jcs.114926 23178947 PMC3654196

[B61] Johnson JO Mandrioli J Benatar M Abramzon Y Van Deerlin VM Trojanowski JQ Gibbs JR Brunetti M Gronka S Wuu J Ding J McCluskey L Martinez-Lage M Falcone D Hernandez DG Arepalli S Chong S Schymick JC Rothstein J Landi F Wang YD Calvo A Mora G Sabatelli M Monsurrò MR Battistini S Salvi F Spataro R Sola P Borghero G Consortium ITALSGEN Galassi G Scholz SW Taylor JP Restagno G Chiò A Traynor BJ Exome sequencing reveals VCP mutations as a cause of familial ALS Neuron 2010 68 857 864 10.1016/j.neuron.2010.11.036 21145000 PMC3032425

[B62] Weihl CC Temiz P Miller SE Watts G Smith C Forman M Hanson PI Kimonis V Pestronk A TDP-43 accumulation in inclusion body myopathy muscle suggests a common pathogenic mechanism with frontotemporal dementia J Neurol Neurosurg Psychiatry 2008 79 1186 1189 10.1136/jnnp.2007.131334 18796596 PMC2586594

[B63] Ishikawa H Yasui K Oketa Y Suzuki M Ono S Increased expression of valosin-containing protein in the skin of patients with amyotrophic lateral sclerosis J Clin Neurosci 2012 19 522 526 10.1016/j.jocn.2011.05.044 22321369

[B64] Kaye FJ Shows TB Assignment of ubiquilin2 (UBQLN2) to human chromosome xp11: 23– > p11.1 by Gene Bridge radiation hybrids Cytogenet Cell Genet 2000 89 116 117 10.1159/000015588 10894951

[B65] Deng H-X Chen W Hong S-T Boycott KM Gorrie GH Siddique N Yang Y Fecto F Shi Y Zhai H Jiang H Hirano M Rampersaud E Jansen GH Donkervoort S Bigio EH Brooks BR Ajroud K Sufit RL Haines JL Mugnaini E Pericak-Vance MA Siddique T Mutations in UBQLN2 cause dominant X-linked juvenile and adult-onset ALS and ALS/dementia Nature 2011 477 211 215 10.1038/nature10353 21857683 PMC3169705

[B66] Daoud H Rouleau GA Motor neuron disease: a role for ubiquilin 2 mutations in neurodegeneration Nat Rev Neurol 2011 7 599 600 10.1038/nrneurol.2011.163 21989241

[B67] Al-Saif A Al-Mohanna F Bohlega S A mutation in sigma-1 receptor causes juvenile amyotrophic lateral sclerosis Ann Neurol 2011 70 913 919 10.1002/ana.22534 21842496

[B68] Morita M Al-Chalabi A Andersen PM Hosler B Sapp P Englund E Mitchell JE Habgood JJ de Belleroche J Xi J Jongjaroenprasert W Horvitz HR Gunnarsson LG Brown RH Jr A locus on chromosome 9p confers susceptibility to ALS and frontotemporal dementia Neurology 2006 66 839 844 10.1212/01.wnl.0000200048.53766.b4 16421333

[B69] Luty AA Kwok JB Dobson-Stone C Loy CT Coupland KG Karlström H Sobow T Tchorzewska J Maruszak A Barcikowska M Panegyres PK Zekanowski C Brooks WS Williams KL Blair IP Mather KA Sachdev PS Halliday GM Schofield PR Sigma nonopioid intracellular receptor 1 mutations cause frontotemporal lobar degeneration-motor neuron disease Ann Neurol 2010 68 639 649 10.1002/ana.22274 21031579

[B70] Hosler BA Siddique T Sapp PC Sailor W Huang MC Hossain A Daube JR Nance M Fan C Kaplan J Hung WY McKenna-Yasek D Haines JL Pericak-Vance MA Horvitz HR Brown RH Jr Linkage of familial amyotrophic lateral sclerosis with frontotemporal dementia to chromosome 9q21-q22 JAMA 2000 284 1664 1669 10.1001/jama.284.13.1664 11015796

[B71] DeJesus-Hernandez M Mackenzie IR Boeve BF Boxer AL Baker M Rutherford NJ Nicholson AM Finch NA Flynn H Adamson J Kouri N Wojtas A Sengdy P Hsiung GY Karydas A Seeley WW Josephs KA Coppola G Geschwind DH Wszolek ZK Feldman H Knopman DS Petersen RC Miller BL Dickson DW Boylan KB Graff-Radford NR Rademakers R Expanded GGGGCC hexanucleotide repeat in noncoding region of C9ORF72 causes chromosome 9p-linked FTD and ALS Neuron 2011 72 245 256 10.1016/j.neuron.2011.09.011 21944778 PMC3202986

[B72] van Blitterswijk M DeJesus-Hernandez M Rademakers R How do C9ORF72 repeat expansions cause amyotrophic lateral sclerosis and frontotemporal dementia: can we learn from other noncoding repeat expansion disorders? Curr Opin Neurol 2012 25 689 700 10.1097/WCO.0b013e32835a3efb 23160421 PMC3923493

[B73] Rademakers R Neumann M Mackenzie IR Advances in understanding the molecular basis of frontotemporal dementia Nat Rev Neurol 2012 8 423 434 22732773 10.1038/nrneurol.2012.117 PMC3629543

[B74] Mori K Weng SM Arzberger T May S Rentzsch K Kremmer E Schmid B Kretzschmar HA Cruts M Van Broeckhoven C Haass C Edbauer D The C9orf72 GGGGCC repeat is translated into aggregating dipeptide-repeat proteins in FTLD/ALS Science 2013 339 1335 1338 10.1126/science.1232927 23393093

[B75] Ash PE Bieniek KF Gendron TF Caulfield T Lin WL Dejesus-Hernandez M van Blitterswijk MM Jansen-West K Paul JW 3rd Rademakers R Boylan KB Dickson DW Petrucelli L Unconventional translation of C9ORF72 GGGGCC expansion generates insoluble polypeptides specific to c9FTD/ALS Neuron 2013 77 639 646 10.1016/j.neuron.2013.02.004 23415312 PMC3593233

[B76] Polymenidou M Lagier-Tourenne C Hutt KR Bennett CF Cleveland DW Yeo GW Misregulated RNA processing in amyotrophic lateral sclerosis Brain Res 2012 1462 3 15 22444279 10.1016/j.brainres.2012.02.059 PMC3707312

[B77] Münch C Sedlmeier R Meyer T Homberg V Sperfeld AD Kurt A Prudlo J Peraus G Hanemann CO Stumm G Ludolph AC Point mutations of the p150 subunit of dynactin (DCTN1) gene in ALS Neurology 2004 63 724 726 10.1212/01.WNL.0000134608.83927.B1 15326253

[B78] Holzbaur ELF Tokito MK Localization of the DCTN1 gene encoding p150 (Glued) to human chromosome 2p13 by fluorescence in situ hybridization Genomics 1996 31 398 399 10.1006/geno.1996.0068 8838327

[B79] Lai C Lin X Chandran J Shim H Yang WJ Cai H The G59S mutation in p150(glued) causes dysfunction of dynactin in mice J Neurosci 2007 27 13982 13990 10.1523/JNEUROSCI.4226-07.2007 18094236 PMC2367233

[B80] Laird FM Farah MH Ackerley S Hoke A Maragakis N Rothstein JD Griffin J Price DL Martin LJ Wong PC Motor neuron disease occurring in a mutant dynactin mouse model is characterized by defects in vesicular trafficking J Neurosci 2008 28 1997 2005 10.1523/JNEUROSCI.4231-07.2008 18305234 PMC6671836

[B81] Puls I Jonnakuty C LaMonte BH Holzbaur EL Tokito M Mann E Floeter MK Bidus K Drayna D Oh SJ Brown RH Jr Ludlow CL Fischbeck KH Mutant dynactin in motor neuron disease Nat Genet 2003 33 455 456 10.1038/ng1123 12627231

[B82] Mitchell J Paul P Chen HJ Morris A Payling M Falchi M Habgood J Panoutsou S Winkler S Tisato V Hajitou A Smith B Vance C Shaw C Mazarakis ND de Belleroche J Familial amyotrophic lateral sclerosis is associated with a mutation in D-amino acid oxidase Proc Natl Acad Sci USA 2010 107 7556 7561 10.1073/pnas.0914128107 20368421 PMC2867752

[B83] Barker RF Hopkinson DA The genetic and biochemical properties of the D-amino acid oxidases in human tissues Ann Hum Genet 1977 41 27 42 10.1111/j.1469-1809.1977.tb01959.x 21608

[B84] Sasabe J Miyoshi Y Suzuki M Mita M Konno R Matsuoka M Hamase K Aiso S D-amino acid oxidase controls motoneuron degeneration through D-serine Proc Natl Acad Sci USA 2012 109 627 632 10.1073/pnas.1114639109 22203986 PMC3258611

[B85] Hayward C Colville S Swingler RJ Brock DJ Molecular genetic analysis of the APEX nuclease gene in amyotrophic lateral sclerosis Neurology 1999 52 1899 1901 10.1212/WNL.52.9.1899 10371543

[B86] Tomkins J Dempster S Banner SJ Cookson MR Shaw PJ Screening of AP endonuclease as a candidate gene for amyotrophic lateral sclerosis (ALS) Neuroreport 2000 11 1695 1697 10.1097/00001756-200006050-00020 10852227

[B87] Vasko MR Guo C Thompson EL Kelley MR The repair function of the multifunctional DNA repair/redox protein APE1 is neuroprotective after ionizing radiation DNA Repair (Amst) 2011 10 942 952 10.1016/j.dnarep.2011.06.004 21741887 PMC3162094

[B88] Skibinski G Parkinson NJ Brown JM Chakrabarti L Lloyd SL Hummerich H Nielsen JE Hodges JR Spillantini MG Thusgaard T Brandner S Brun A Rossor MN Gade A Johannsen P Sørensen SA Gydesen S Fisher EM Collinge J Mutations in the endosomal ESCRTIII-complex subunit CHMP2B in frontotemporal dementia Nat Genet 2005 37 806 808 10.1038/ng1609 16041373

[B89] Cox LE Ferraiuolo L Goodall EF Heath PR Higginbottom A Mortiboys H Hollinger HC Hartley JA Brockington A Burness CE Morrison KE Wharton SB Grierson AJ Ince PG Kirby J Shaw PJ Mutations in CHMP2B in lower motor neuron predominant amyotrophic lateral sclerosis (ALS) PLoS One 2010 5 e9872 10.1371/journal.pone.0009872 20352044 PMC2844426

[B90] Belly A Bodon G Blot B Bouron A Sadoul R Goldberg Y CHMP2B mutants linked to frontotemporal dementia impair maturation of dendritic spines J Cell Sci 2010 123 2943 2954 10.1242/jcs.068817 20699355 PMC3013364

[B91] Ghazi-Noori S Froud KE Mizielinska S Powell C Smidak M Fernandez De Marco M O’Malley C Farmer M Parkinson N Fisher EM Asante EA Brandner S Collinge J Isaacs AM Progressive neuronal inclusion formation and axonal degeneration in CHMP2B mutant transgenic mice Brian 2012 135 Pt 3 819 832 10.1093/brain/aws006 22366797

[B92] Al-Chalabi A Andersen PM Nilsson P Chioza B Andersson JL Russ C Shaw CE Powell JF Leigh PN Deletions of the heavy neurofilament subunit tail in amyotrophic lateral sclerosis Hum. Molec. Genet 1999 8 157 164 10.1093/hmg/8.2.157 9931323

[B93] Couillard-Després S Zhu Q Wong PC Price DL Cleveland DW Julien JP Protective effect of neurofilament heavy gene overexpression in motor neuron disease induced by mutant superoxide dismutase Proc Natl Acad Sci USA 1998 95 9626 9630 10.1073/pnas.95.16.9626 9689131 PMC21389

[B94] Mersiyanova IV Perepelov AV Polyakov AV Sitnikov VF Dadali EL Oparin RB Petrin AN Evgrafov OV A new variant of Charcot-Marie-Tooth disease type 2 is probably the result of a mutation in the neurofilament-light gene Am J Hum Gene 2000 67 37 46 10.1086/302962 PMC1287099 10841809

[B95] Skvortsova V Shadrina M Slominsky P Levitsky G Kondratieva E Zherebtsova A Levitskaya N Alekhin A Serdyuk A Limborska S Analysis of heavy neurofilament subunit gene polymorphism in Russian patients with sporadic motor neuron disease (MND) Eur J Hum Genet 2004 12 241 244 10.1038/sj.ejhg.5201144 14722583

[B96] Giordano G Cole TB Furlong CE Costa LG Paraoxonase 2 (PON2) in the mouse central nervous system: a neuroprotective role? Toxicol Appl Pharmacol 2011 256 369 378 10.1016/j.taap.2011.02.014 21354197 PMC3155737

[B97] Horner RD Kamins KG Feussner JR Grambow SC Hoff-Lindquist J Harati Y Mitsumoto H Pascuzzi R Spencer PS Tim R Howard D Smith TC Ryan MA Coffman CJ Kasarskis EJ Occurrence of amyotrophic lateral sclerosis among Gulf War veterans Neurology 2003 61 742 749 10.1212/01.WNL.0000069922.32557.CA 14504315

[B98] Saeed M Siddique N Hung WY Usacheva E Liu E Sufit RL Heller SL Haines JL Pericak-Vance M Siddique T Paraoxonase cluster polymorphisms are associated with sporadic ALS Neurology 2006 67 771 776 10.1212/01.wnl.0000227187.52002.88 16822964

[B99] Valdmanis PN Kabashi E Dyck A Hince P Lee J Dion P D’Amour M Souchon F Bouchard JP Salachas F Meininger V Andersen PM Camu W Dupré N Rouleau GA Association of paraoxonase gene cluster polymorphisms with ALS in France, Quebec, and Sweden Neurology 2008 71 514 520 10.1212/01.wnl.0000324997.21272.0c 18695162

[B100] Ticozzi N LeClerc AL Keagle PJ Glass JD Wills AM van Blitterswijk M Bosco DA Rodriguez-Leyva I Gellera C Ratti A Taroni F McKenna-Yasek D Sapp PC Silani V Furlong CE Brown RH Jr Landers JE Paraoxonase gene mutations in amyotrophic lateral sclerosis Ann Neurol 2010 68 102 107 10.1002/ana.21993 20582942 PMC2945725

[B101] Mizuno Y Fujita Y Takatama M Okamoto K Peripherin partially localizes in Bunina bodies in amyotrophic lateral sclerosis J Neurol Sci 2011 302 14 18 10.1016/j.jns.2010.12.023 21241994

[B102] Beaulieu JM Nguyen MD Julien JP Late onset of motor neurons in mice overexpressing wild-type peripherin J Cell Biol 1999 147 531 544 10.1083/jcb.147.3.531 15132161 PMC2151189

[B103] Corrado L Carlomagno Y Falasco L Mellone S Godi M Cova E Cereda C Testa L Mazzini L D’Alfonso S A novel peripherin gene (PRPH) mutation identified in one sporadic amyotrophic lateral sclerosis patient Neurobiol Aging 2011 32 552 e1 e6 20363051 10.1016/j.neurobiolaging.2010.02.011

[B104] Robertson J Doroudchi MM Nguyen MD Durham HD Strong MJ Shaw G Julien J-P Mushynski WE A neurotoxic peripherin splice variant in a mouse model of ALS J Cell Biol 2003 160 939 949 10.1083/jcb.200205027 12642616 PMC2173778

[B105] Schwab C Yu S McGeer EG McGeer PL Optineurin in Huntington’s disease intranuclear inclusions Neurosci Lett 2012 506 149 154 10.1016/j.neulet.2011.10.070 22085693

[B106] Xiao S Tjostheim S Sanelli T McLean JR Horne P Fan Y Ravits J Strong MJ Robertson J An aggregate-inducing peripherin isoform generated through intron retention is upregulated in amyotrophic lateral sclerosis and associated with disease pathology J Neurosci 2008 28 1833 1840 10.1523/JNEUROSCI.3222-07.2008 18287500 PMC6671437

[B107] Lefebvre S Burlet P Liu Q Bertrandy S Clermont O Munnich A Dreyfuss G Melki J Correlation between severity and SMN protein level in spinal muscular atrophy Nat Genet 1997 16 265 269 10.1038/ng0797-265 9207792

[B108] Veldink JH Kalmijn S Van der Hout AH Lemmink HH Groeneveld GJ Lummen C Scheffer H Wokke JH Van den Berg LH SMN genotypes producing less SMN protein increase susceptibility to and severity of sporadic ALS Neurology 2005 65 820 825 10.1212/01.wnl.0000174472.03292.dd 16093455

[B109] Corcia P Mayeux-Portas V Khoris J de Toffol B Autret A Muh JP Camu W Andres C French ALS Research Group Amyotrophic Lateral Sclerosis.Abnormal SMN1 gene copy number is a susceptibility factor for amyotrophic lateral sclerosis Ann Neurol 2002 51 243 246 10.1002/ana.10104 11835381

[B110] Oosthuyse B Moons L Storkebaum E Beck H Nuyens D Brusselmans K Van Dorpe J Hellings P Gorselink M Heymans S Theilmeier G Dewerchin M Laudenbach V Vermylen P Raat H Acker T Vleminckx V Van Den Bosch L Cashman N Fujisawa H Drost MR Sciot R Bruyninckx F Hicklin DJ Ince C Gressens P Lupu F Plate KH Robberecht W Herbert JM Collen D Carmeliet P Deletion of the hypoxia-response element in the vascular endothelial growth factor promoter causes motor neuron degeneration Nat Genet 2001 28 131 138 10.1038/88842 11381259

[B111] Storkebaum E Treatment of motoneuron degeneration by intracerebroventricular delivery of VEGF in a rat model of ALS Nat Neurosci 2005 8 85 92 10.1038/nn1360 15568021

[B112] Brockington A Expression of vascular endothelial growth factor and its receptors in the central nervous system in amyotrophic lateral sclerosis J Neuropathol Exp Neurol 2006 65 26 36 10.1097/01.jnen.0000196134.51217.74 16410746

[B113] Lambrechts D Poesen K Fernández-Santiago R Al-Chalabi A Del Bo R Van Vught PW Khan S Marklund SL Brockington A van Marion I Anneser J Shaw C Ludolph AC Leigh NP Comi GP Gasser T Shaw PJ Morrison KE Andersen PM Van den Berg LH Thijs V Siddique T Robberecht W Carmeliet P Meta-analysis of vascular endothelial growth factor variations in amyotrophic lateral sclerosis: increased susceptibility in male carriers of the -2578AA genotype J Med Genet 2009 46 840 846 10.1136/jmg.2008.058222 18413368

[B114] Baker M Mackenzie IR Pickering-Brown SM Gass J Rademakers R Lindholm C Snowden J Adamson J Sadovnick AD Rollinson S Cannon A Dwosh E Neary D Melquist S Richardson A Dickson D Berger Z Eriksen J Robinson T Zehr C Dickey CA Crook R McGowan E Mann D Boeve B Feldman H Hutton M Mutations in progranulin cause tau-negative frontotemporal dementia linked to chromosome 17 Nature 2006 442 916 919 10.1038/nature05016 16862116

[B115] Schymick JC Yang Y Andersen PM Vonsattel JP Greenway M Momeni P Elder J Chiò A Restagno G Robberecht W Dahlberg C Mukherjee O Goate A Graff-Radford N Caselli RJ Hutton M Gass J Cannon A Rademakers R Singleton AB Hardiman O Rothstein J Hardy J Traynor BJ Progranulin mutations and amyotrophic lateral sclerosis or amyotrophic lateral sclerosis-frontotemporal dementia phenotypes J Neurol Neurosurg Psychiatry 2007 78 754 756 17371905 10.1136/jnnp.2006.109553 PMC2117704

[B116] Elden AC Kim HJ Hart MP Chen-Plotkin AS Johnson BS Fang X Armakola M Geser F Greene R Lu MM Padmanabhan A Clay-Falcone D McCluskey L Elman L Juhr D Gruber PJ Rüb U Auburger G Trojanowski JQ Lee VM Van Deerlin VM Bonini NM Gitler AD Ataxin-2 intermediate-length polyglutamine expansions are associated with increased risk for ALS Nature 2010 466 1069 1075 10.1038/nature09320 20740007 PMC2965417

[B117] Ralser M Nonhoff U Albrecht M Lengauer T Wanker EE Lehrach H Krobitsch S Ataxin-2 and huntingtin interact with endophilin-A complexes to function in plastin-associated pathways Hum Molec Genet 2005 14 2893 2909 10.1093/hmg/ddi321 16115810

